# Effects of low frequency repetitive transcranial magnetic stimulation on motor recovery in subacute stroke patients with different motor evoked potential status: a randomized controlled trial

**DOI:** 10.3389/fneur.2024.1460925

**Published:** 2024-10-17

**Authors:** Wenjun Qian, Xiaoyu Liao, Xiaowen Ju, Yaxin Gao, Miao Wu, Chen Xie, Yaoying Zhang, Xianming Long, Surong Qian, Yan Gong

**Affiliations:** ^1^Department of Rehabilitation Medicine, Gusu School, The Affiliated Suzhou Hospital of Nanjing Medical University, Suzhou Municipal Hospital, Nanjing Medical University, Suzhou, China; ^2^Department of Rheumatology and Immunology, The Affiliated Suzhou Hospital of Nanjing Medical University, Suzhou Municipal Hospital, Gusu School, Nanjing Medical University, Suzhou, China

**Keywords:** stroke, transcranial magnetic stimulation, motor evoked potential, motor recovery, cortical excitability

## Abstract

**Objectives:**

To explore the effects of low-frequency repetitive transcranial magnetic stimulation (LF-rTMS) on motor function and cortical excitability in stroke patients with different motor evoked potential (MEP) status.

**Methods:**

A total of 80 stroke patients were enrolled in this randomized controlled trial and divided into two groups according to MEP status (− or +) of lesioned hemisphere. Then, each group was randomly assigned to receive either active or sham LF-rTMS. In addition to conventional rehabilitation, all participants received 20 sessions of rTMS at 1 Hz frequency through the active or the sham coil over 4 weeks. Fugl-Meyer Assessment (FMA), National Institutes of Health Stroke Scale (NIHSS), Shoulder Abduction Finger Extension (SAFE) and Barthel Index (BI), bilateral resting motor threshold (rMT), amplitude of Motor evoked potential (MEP) and Central Motor Conduction Time (CMCT), and Interhemispheric asymmetry (IHA) were blindly assessed at baseline, 4 weeks and 8 weeks after treatment, respectively.

**Results:**

At 4 weeks after intervention, FMA and NIHSS changed scores in 1 Hz MEP(+) group were significantly higher than those in the other three groups (*p* < 0.001). After receiving 1 Hz rTMS, stroke patients with MEP(+) showed significant changes in their bilateral cortical excitability (*p* < 0.05). At 8 weeks after intervention, 1 Hz MEP(+) group experienced higher changes in NIHSS, FMA, SAFE, and BI scores than other groups (*p* < 0.001). Furthermore, 1 Hz rTMS intervention could decrease unaffected cortical excitability and enhance affected cortical excitability of stroke patients with MEP(+) (*p* < 0.05). The correlation analysis revealed that FMA motor change score was associated with decreased unaffected MEP amplitude (*r* = −0.401, *p* = 0.010) and decreased affected rMT (*r* = −0.584, *p* < 0.001) from baseline, which was only observed in the MEP(+) group.

**Conclusion:**

The effects of LF-rTMS on motor recovery and cortical excitability were more effective in stroke patients with MEP than those with no MEP.

## Introduction

Stroke is a prevalent neurological disease with high disability and mortality (1). Stroke is the second leading cause of mortality worldwide, with nearly 7 million deaths, and is the third leading cause of disability globally (2). In 2020, there were more than 17.8 million stroke patients, 3.4 million new cases of stroke, and 2.3 million stroke deaths among people aged 40 or older in China (3). Stroke often causes a variety of neurologic deficits, including motor dysfunction. In addition, motor dysfunction is a frequent neurological dysfunction after stroke and an important contributory factor to a patient’s ability to live independently (4). In generally, the spontaneous neurobehavioral recovery in stroke patients often follow a logical pattern of reaching a plateau in the first 10 weeks after stroke (5). Patients with stroke often suffer from limited activities of daily living and restricted participation due to motor impairment. Despite undergoing intensive rehabilitation therapies, most stroke survivors still suffer from motor impairment (6). Therefore, novel neurorehabilitation strategies are required to promote motor recovery in stroke patients.

Repetitive transcranial magnetic stimulation (rTMS) is one of noninvasive brain stimulation (NIBS) and has been extensively investigated for motor recovery after stroke as a neural electrophysiological stimulation technique (7). Based on the interhemispheric inhibition (IHI) model, low-frequency rTMS (LF-rTMS) over the contralesional primary motor cortex (M1) could normalize imbalanced interhemispheric inhibition by suppressing the contralesional M1 and then promote motor function recovery after stroke (7). However, some studies reported that LF-rTMS over the contralesional M1 did not significantly improve motor recovery in patients with subacute stroke (8) and those with severe hemiplegia (9, 10). Although the IHI theoretical model is widely accepted as a recommendation for the application of rTMS in motor recovery after stroke, it is not applicable to all patients, especially those with severe motor impairment (11, 12). The motor evoked potential (MEP) evoked by TMS serves as an indicator of the functional corticospinal tract (CST) (13, 14). Compared to functional magnetic resonance imaging (fMRI), MEP can be conveniently and routinely tested in stroke patients. Currently, MEP has been studied to evaluate brain injury severity and predict motor recovery in stroke patients (13, 15). However, the connection between MEP status and the therapeutic effects of LF-rTMS remains unclear (9, 13, 16).

Therefore, this research aimed to investigate the effects of LF-rTMS on motor function and cortical excitability in stroke patients with different MEP status, which would guide the clinical application of rTMS. In this study, considering that the optimal time window for neuroplasticity is the subacute phase after stroke (17), we recruited patients with the subacute phase of stroke. We hypothesized that LF-rTMS over the contralesional M1 would produce greater motor function recovery in subacute stroke patients with MEP than in patients with no MEP.

## Methods

This study was a randomized, controlled, prospective, single-blinded clinical trial. Participants with stroke were recruited from the Rehabilitation medicine department of our hospital. The following criteria had to be met for inclusion: (1) a first unilateral stroke within the region of the middle cerebral artery as identified by MRI (18), (2) the patient’s age ranged from 30 to 80 years, and (3) the patient’s duration of stroke was within 1–3 months of its onset. The following patients were excluded: (1) those with limb dysfunction caused by other nervous system diseases, (2) those with a history of seizures, (3) those with severe cognitive impairment (Mini-mental state examination ≤ 9 scores) or severe aphasia dysfunction (Boston diagnostic aphasia examination ≤ 1 grade), (4) those who were contraindicated for TMS, such as epilepsy, metal implants, and pregnancy, (5) those who used benzodiazepines or antidepressants, and (6) those who used muscle relaxants. This study protocol was approved by the Institutional Review Board of our hospital. All participants provided informed consent prior to participation. Each patient was informed about the possibility of being randomized to the sham rTMS group.

**Figure 1:**
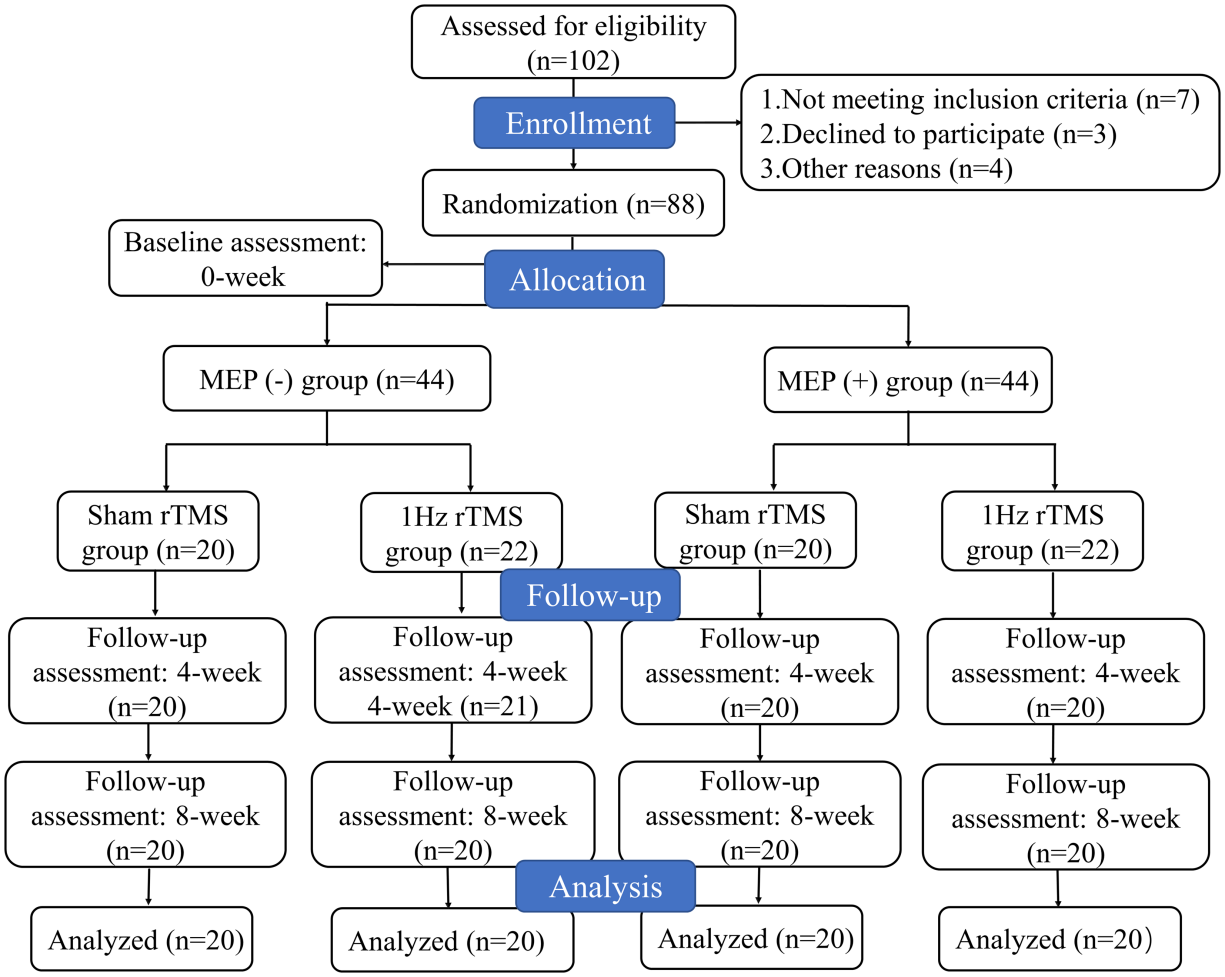
Flow chart of the study shows the patient enrollment, intervention, and analysis.

Stroke patients were divided into two groups according to MEP status of lesioned hemisphere, including MEP (−) (patients who did not show MEP) and MEP (+) (patients who showed MEP) groups. Then, each group was randomly assigned to receive either active or sham LF-rTMS. Therefore, participants were assigned to one of four groups: sham MEP (−), 1 Hz MEP (−), sham MEP (+), and 1 Hz MEP (+) groups. The MEP was obtained from bilateral abductor pollicis brevis muscles (APB) in a relaxed state and the single pulse TMS stimulation of affected APB was used to evaluate MEP status (19). When there was a slight voluntary muscle contraction, the MEPs was recorded. If the patient was unable to perform the affected hand or arm muscle, they were asked to activate the corresponding muscle on the unaffected side. MEP(+) was defined as a minimum peak-to-peak amplitude of 200 μV for at least two of the three responses (20). A study coordinator who wasn’t involved in the screening handled the randomization procedure for patients receiving either active or sham LF-rTMS. The opaque envelopes with sequential numbers were used to store the randomized blocks, and the order of randomization was produced by a computer. The blinding of patients and intervention providers cannot be maintained due to the specificity of this experiment. Nonetheless, the study’s statisticians and evaluators continued to be blinded. This study had following phases: (1) randomization and baseline assessment (T0), (2) assessment after the fourth week of treatment (T1), (3) follow-up 8 weeks after treatment (T2). The participant flowchart is illustrated in Figure 1.

Patients underwent real or sham 1 Hz rTMS protocol administered using the MagproR30 magnetic stimulation instrument produced by Magventure Company in Denmark. Before rTMS treatment, a detailed risk assessment and emergency management measures were conducted. Wearing earplugs and other hearing protection devices were also recommended. Additionally, the hotspots over the primary motor cortex (M1) in both hemispheres were identified in the baseline assessment session and an electroencephalogram cap was worn for tracer marking during treatment. In real rTMS, the coil was placed tangentially in a posterior anterior plane over the motor representation. The contralesional M1 was stimulated in each session for 20 min at a frequency of 1 Hz and an intensity of 100% of the patient’s contralesional rMT, achieving 1,200 stimuli. The stimulation duration was 4 weeks. In sham rTMS, the coil was perpendicular to the scalp on contralesional M1 with the same intensity and frequency as that in real rTMS. All participants received the same intensity of conventional physical rehabilitation and occupational therapy after the intervention, which mainly included muscle stretching, passive-assisted motor training, neuromuscular facilitation training, and task-oriented training. Each training lasted approximately 40 min, once a day, 5 days a week, continuous training for 4 weeks, conducted by the same therapists blinded to group allocation. Moreover, all participants completed the identical routine exercise training after sham or 1 Hz rTMS treatment.

All assessments were conducted by two therapists blinded to group allocation at baseline, 4 weeks, and 8 weeks after treatment. Demographic data such as gender, age, time since stroke, dominant hand, type of stroke, and stroke location were collected at baseline. The assessment of bilateral cortical excitability and motor function were the primary outcome measures. Motor function was measured by using the Fugl-Meyer Assessment (FMA) (21), the National Institutes of Health Stroke Scale (NIHSS) (22), Shoulder Abduction Finger Extension (SAFE) (13), and Barthel Index (BI).

Bilateral motor cortical excitability was assessed through single-pulse TMS in both hemispheres, including rMT, amplitude of MEP, central motor conduction time (CMCT), and interhemispheric asymmetry (IHA) (16, 19, 23). In this study, we adopted magnetic stimulation electromyography (EMG) attachment MEP monitor of single-channel EMG acquisition, sampling 100ks/s 16bit, and selected the APB as the target muscle by stimulating the central motor area. Then, the Magpro family software was used for data collection and analysis. Firstly, the rMT was defined as the lowest stimulation intensity that produced at least 50 μv of MEP for at least 5 of 10 consecutive stimulations through EMG recording (24, 25). Secondly, the MEP amplitude was measured by recording 10 averaged MEPs evoked from the M1 hotspot by using a stimulation intensity of 120% rMT measured at baseline (24). Thirdly, the CMCT refers to the time from cerebral cortex to spinal cord after single pulse TMS stimulation. Magnetic stimuli were applied over the lower cervical spine to measure peripheral conduction time. Then, CMCT values of bilateral hemispheres were calculated by subtracting the peripheral motor conduction time from the shortest corticomotor latency (9). Finally, the IHA was defined as the ratio of paretic MEP amplitude minus non-paretic MEP amplitude to the sum of both hemispherical MEP amplitudes (16).

The statistical software package SPSS 24.0 version was used for statistical analysis of the data. The measurement data with normal distribution and homogeneity of variance assumptions were expressed as mean ± standard deviation. Data were then analyzed separately using repeated-measures and multivariate ANOVA with between-subjects factor group, and within-subjects factor time as well as multiple comparisons with LSD corrections. In addition, the measurement data and grade data with non-normal distribution were expressed by the median (interquartile range), and independent-samples Kruskal-Wallis test was used to compare these data. The correlation analysis was conducted to identify whether there were correlations between motor improvement and changes in cortical excitability at 8 weeks follow-up after interventions. A difference that was deemed statistically significant was defined as a *p* value less than 0.05.

## Results

A total of 102 patients underwent eligibility screening. Seven patients were excluded because they did not meet the requirements for inclusion. Three patients declined to participate in the study. In addition, four patients were also excluded because they were unable to tolerate rTMS intervention and were lost to follow-up. Ultimately, only 88 patients were included in this study. The participants’ flowchart is shown in Figure 1. The patients’ baseline characteristics with MEP and no MEP were displayed in Tables 1, 2 respectively. Four groups were well matched on basic demographics and variables, including age, gender, and cognitive function. There were no significant differences in baseline demographic variables or outcome measures between the sham and 1 Hz rTMS groups under different MEP conditions. Furthermore, apart from five transient head headaches at the start of the stimulation, no side effects such as seizures were observed in this study.

**Table 1:** Characteristic stroke patients with no MEP.

Variables	Sham MEP(−)	1 Hz MEP(−)	*p* value
*N*	20	20	
Gender (male/female)	15/5	14/6	0.723
Age (years)	59.65 ± 14.45	60.35 ± 12.77	0.872
Time since stroke (days)	47.75 ± 12.30	43.05 ± 11.74	0.224
Dominant hand (right/left)	19/1	18/2	0.548
Type of stroke (infract/hemorrhage)	14/6	15/5	0.723
Paretic limb (left/right)	11/9	11/9	1.000
Stroke location (subcortical/cortical involvement) (n)	17/3	16/4	0.548
NIHSS	9.60 ± 1.57	10.05 ± 2.18	0.459
SAFE	2.00 ± 0.92	2.05 ± 0.60	0.587
FMA motor scores	24.55 ± 4.83	23.60 ± 4.07	0.506
BI	33.80 ± 6.76	34.45 ± 4.61	0.725
rMT (unaffected) (%)	36.80 ± 8.04	37.75 ± 11.22	0.760
MEP amplitude (unaffected) (uv)	715.2 (464.2–891.3)	742.2 (662.1–1156.0)	0.102
CMCT (unaffected) (ms)	7.54 ± 0.87	7.66 ± 0.83	0.646

MEP, Motor evoked potential; MEP(−), Patients who showed no MEP; NIHSS, National Institute of Health stroke scale; FMA, Fugl-Meyer assessment; BI, Barthel index; rMT, Resting motion threshold; MEP, Motor evoked potential; CMCT, Central motor conduction time.

**Table 2:** Characteristic stroke patients with MEP.

Variables	Sham MEP(+)	1 Hz MEP(+)	*p*-values
*N*	20	20	
Gender (male/female)	17/3	13/7	0.144
Age (years)	61.55 ± 10.67	59 ± 9.35	0.427
Time since stroke (days)	43.15 ± 11.19	42.75 ± 10.62	0.491
Dominant hand (right/left)	20/0	19/1	0.311
Type of stroke (infract/hemorrhage)	14/6	13/7	0.736
Paretic limb (left/right)	10/9	11/9	0.752
Stroke location (subcortical/cortical involvement) (n)	18/2	18/2	1.000
NIHSS	7.70 ± 1.49	7.6 ± 1.78	0.849
SAFE	3.55 ± 0.82	3.40 ± 0.59	0.515
FMA motor scores	36.10 ± 6.56	38.35 ± 7.07	0.304
BI	52.75 ± 9.79	53.40 ± 6.41	0.805
rMT (unaffected) (%)	44.95 ± 7.57	43.45 ± 6.61	0.481
MEP amplitude (unaffected) (uV)	574.0 (448.4–936.1)	563.5 (356.9–947.6)	0.655
MEP latency (unaffected) (ms)	24.70 ± 1.54	24.89 ± 2.24	0.761
CMCT (unaffected) (ms)	7.92 ± 1.03	8.13 ± 0.99	0.503
rMT (affected) (%)	69.25 ± 9.77	70.45 ± 12.08	0.732
MEP amplitude (affected) (uV)	222.3 (175.20–252.97)	246.8 (162.4–294.62)	0.441
CMCT (affected) (ms)	12.74 ± 1.69	13.08 ± 1.21	0.466
IHA	−0.45 ± 0.20	−0.42 ± 0.21	0.663

MEP, Motor evoked potential; MEP(+), Patients who showed MEP; NIHSS, National Institute of Health stroke scale; FMA, Fugl-Meyer assessment; BI, Barthel index; rMT, Resting motion threshold; MEP, Motor evoked potential; CMCT, Central motor conduction time; IHA, Interhemispheric asymmetry.

**Figure 2:**
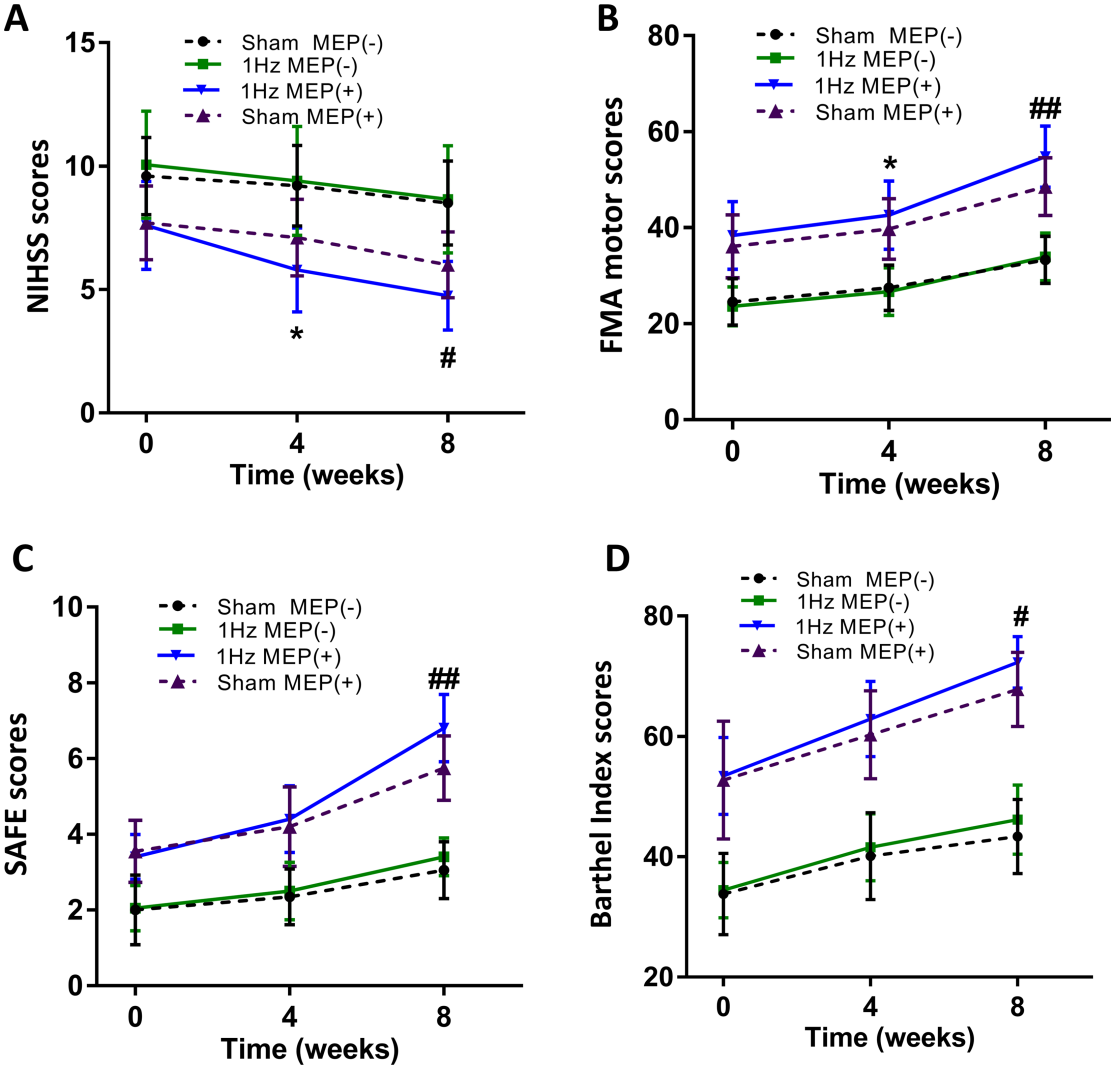
Changes in mean rating scores of motor function indexes from baseline to 8 weeks. **(A)** NIHSS scores, **(B)** FMA motor scores and unaffected rMT, **(C)** SAFE scores, **(D)** Barthel Index scores.

At four-week follow-up after the intervention, motor function and cortical excitability data before and after the intervention are shown in Table 3. These data showed differential changes in outcome measures of motor function and cortical excitability in four groups. Regarding motor performance, there were no significant differences in motor function and cortical excitability between the sham MEP(−) and 1 Hz MEP(−) groups (all *p >* 0.05). Compared with the sham MEP(+) group, the NIHSS values of 1 Hz MEP(+) group decreased obviously (*t* = 2.522, *p* = 0.016), while the FMA values of 1 Hz MEP(+) group increased significantly (*t* = −2.298, *p* = 0.027). Furthermore, NIHSS and FMA scores between sham MEP(+) and 1 Hz MEP(+) groups changed significantly (*t* = 7.397, *p <* 0.001, and *t* = −5.983, *p <* 0.001, respectively). Moreover, the 1 Hz MEP(+) group resulted in considerably greater changes in NIHSS, FMA, SAFE, and BI scores than the 1 Hz MEP(−) group after 1 Hz rTMS treatment (all *p* < 0.05). However, there were no different changes in motor function in the MEP(−) group after 1 Hz rTMS treatment, including the scores of NIHSS, FMA, SAFE, and BI (all *p* > 0.05) (Figure 2).

**Table 3:** Changes in motor function and cortical excitability between baseline and 4 weeks.

Variables	Sham MEP(−)	1 Hz MEP(−)	Sham MEP(+)	1 Hz MEP(+)		
Motor function	HR (95%CI)	HR (95%CI)	HR (95%CI)	HR (95%CI)	Statistic value	*p* value
NIHSS	−0.40 (−0.63, −0.16)	−0.65 (−0.92, −0.37)	−0.60 (−0.83, −0.36)	−1.80 (−2.04, −1.55)^b^^,^^c^	28.641	<0.001
FMA motor scores	2.90 (2.39, 3.40)	3.10 (2.40, 3.79)	3.60 (2.91, 4.28)	6.25 (5.62, 6.87)^b^^,^^c^	26.541	<0.001
SAFE	0.35 (0.12, 0.58)	0.45 (0.21, 0.69)	0.65 (0.27, 1.03)	1.00 (0.66, 1.34)^b^	3.903	0.012
BI	6.30 (4.85, 7.74)	7.10 (5.93, 8.26)	7.50 (5.56, 9.43)	9.50 (8.45, 10.54)^b^	3.913	0.012
**Cortical excitability**						
rMT (unaffected) (%)	1.50 (0.94, 2.06)	2.00 (1.54, 2.45)	1.40 (0.766, 2.03)	2.75 (1.97, 3.52)^c^	4.398	0.007
MEP amplitude (unaffected) (uv)	−13.40 (−26.62, −8.52)	−26.45 (−119.95, −12.75)	−14.20 (−22.21, −7.65)	−29.80 (−63.75, −12.25)^c^	10.713	0.013
CMCT (unaffected) (ms)	0.46 (0.35, 0.57)	0.77 (0.54, 1.01)	0.59 (0.41, 0.78)	1.04 (0.63, 1.44)^c^	4.178	0.009
rMT (affected) (%)	–	–	−11.9 (−13.79, −10.00)	−18.15 (−21.86, −14.43)^c^	3.135	0.003
MEP amplitude (affected) (uv)	–	–	21 (11.92, 37.10)	55.5 (46.27, 71.35)^c^	−3.571	<0.001
CMCT (affected) (ms)	–	–	−0.81 (−1.05, −0.56)	−2.19 (−2.58, −1.79)^c^	6.169	<0.001
IHA	–	–	0.06 (−1.11, −0.02)	0.13 (−1.11, −0.02)^c^	−3.021	0.004

a The *p*-values of Sham MEP(+) vs Sham MEP(−) > 0.05.

b The *p*-value of 1 Hz MEP(+) vs 1 Hz MEP(−) < 0.05.

c The *p*-value of 1 Hz MEP(+) vs Sham MEP(+) < 0.05.

d The *p*-values of 1 Hz MEP(−) vs Sham MEP(−) > 0.05.

MEP, Motor evoked potential; MEP(−), The absence of MEP; MEP(+), The presence of MEP; NIHSS, National Institute of Health stroke scale; FMA, Fugl-Meyer assessment; BI, Barthel index; rMT, Resting motor threshold; CMCT, Central motor conduction time; IHA, Interhemispheric asymmetry.

There was no significant change in contralesional cortical excitability between sham MEP(−) and 1 Hz MEP(−) groups at the four-week follow-up. Compared to the sham MEP(+) group, the 1 Hz MEP(+) group showed prolonged contralateral CMCT (*t* = −2.293, *p* = 0.027), increased ipsilesional MEP amplitude (*z* = −2.259, *p* = 0.024), and a shortened ipsilesional CMCT (*t* = −2.485, *p* = 0.017). Furthermore, there were significant changes in bilateral cortical excitability of patients with MEP(+) after 4-week of 1 Hz rTMS treatment. Compared with the 1 Hz MEP(−) group, the contralateral rMT value of the 1 Hz MEP(+) group was significantly increased (*z* = −2.357, *p* = 0.018), and contralateral CMCT was prolonged (*t* = −2.660, *p* = 0.011). However, no significant changes in the cortical excitability of bilateral hemispheres were observed in these patients with no MEP after 4-week of 1 Hz rTMS treatment (Table 3).

At eight-week follow-up after the interventions, the pre-to post-intervention data of motor function and cortical excitability were presented in Table 4. Moreover, the changes in the outcome measures of motor function and cortical excitability of four groups were also presented. Regarding motor performance, after administration of 1 Hz rTMS to patients with MEP(−), there were no significant changes in the motor function and cortical excitability indices of these patients with MEP(−) (all *p* > 0.05). After administration of 1 Hz TMS to patients with MEP(+), the 1 Hz MEP(+) group had lower NIHSS scores (*t* = 2.430, *p* = 0.020), higher SAFE scores (*t* = −3.804, *p* = 0.001) and FMA scores (*t* = −3.197, *p* = 0.003) than the sham MEP(+). Furthermore, the BI scores of 1 Hz MEP(+) group obviously increased (*t* = −2.677, *p* = 0.011). On the other hand, the changes in NIHSS, FMA, SAFE, and BI scores were greater in the 1 Hz MEP(+) group than in the other three groups (all *p* < 0.01), which were illustrated in Figure 2.

**Table 4:** Changes in motor function and cortical excitability between baseline and 8 weeks.

Variables	Sham MEP(−)	1 Hz MEP(−)	Sham MEP(+)	1 Hz MEP(+)		
Motor function	HR (95%CI)	HR (95%CI)	HR (95%CI)	HR (95%CI)	Stastistic value	*p* value
NIHSS	−1.10 (−1.64, −0.55)	−1.40 (−1.75, −1.04)	−1.70 (−1.96, −1.43)^a^	−2.65 (−3.15, −2.14)^b^^,^^c^	10.481	<0.001
FMA motor scores	8.75 (7.97, 9.52)	10.30 (8.91, 11.68)	12.45 (11.23, 13.66)^a^	16.45 (15.09, 17.80)^b^^,^^c^	33.511	<0.001
SAFE	1.05 (0.76, 1.33)	1.35 (1.00, 1.69)	2.20 (1.78, 2.61)^a^	3.40 (3.04, 3.75)^b^^,^^c^	38.728	<0.001
BI	9.55 (7.91, 11.18)	11.70 (10.00, 13.39)	15.05 (12.61, 17.48)^a^	18.90 (16.89, 20.90)^b^^,^^c^	18.868	<0.001
**Cortical excitability**						
rMT (unaffected) (%)	3.50 (2.57, 4.43)	4.75 (3.35, 5.96)	3.65 (2.48, 4.82)	5.90 (4.92, 6.87)^c^	4.695	0.005
MEP amplitude (unaffected) (uv)	−33.0 (−45.30, −23.35)	−46.20 (−192.10, −36.65)	−26.45 (−36.25, −19.32)^a^	−51.30 (−74.25, −39.85)^c^	6.317	0.001
CMCT (unaffected) (ms)	0.60 (0.40, 0.80)	0.69 (0.52, 0.85)	0.79 (0.50, 1.08)	1.26 (0.69, 1.83)^b^^,^^c^	3.264	0.026
rMT (affected) (%)	–	–	−14.4 (−16.38, −12.41)	−23.7 (−18.75, −15.32)^c^	4.531	<0.001
MEP amplitude (affected) (uv)	–	–	114.05 (75.02–127.60)	144.5 (113.32, 213.42)^c^	−3.327	0.004
CMCT (affected) (ms)	–	–	−1.98 (−2.33, −1.62)	−3.35 (−3.84, −2.86)^c^	4.728	<0.001
IHA	–	–	0.18 (−0.27, −0.08)	0.36 (−0.27, −0.07)^c^	3.713	0.001

a The *p*-value of Sham MEP(+) vs Sham MEP(−) < 0.05.

b The *p*-value of 1 Hz MEP(+) vs 1 Hz MEP(−) < 0.05.

c The *p*-value of 1 Hz MEP(+) vs Sham MEP(+) < 0.05.

d The *p*-values of 1 Hz MEP(−) vs Sham MEP(−) > 0.05.

MEP, Motor evoked potential; MEP(−), The absence of MEP; MEP(+), The presence of MEP; NIHSS, National Institute of Health stroke scale; FMA, Fugl-Meyer assessment; BI: Barthel index; rMT, Resting motor threshold; CMCT, Central motor conduction time; IHA, Interhemispheric asymmetry.

As for the cortical excitability at the eight-week follow-up after the intervention, there was still no obvious change in the cortical excitability of the contralateral side between the sham MEP (−) group and 1 Hz MEP (−) group. Compared to the sham MEP(+) group, the 1 Hz MEP(+) group manifested decreased ipsilesional rMT (*t* = 2.667, *p* = 0.011), increased ipsilesional MEP amplitude (*t* = −2.945, *p* = 0.005), shortened ipsilesional CMCT (*t* = 2.566, *p* = 0.014), and decreased IHA (*t* = −2.877, *p* = 0.007) at 8-week follow-up. On the other hand, there were significant changes in the indices of bilateral cortical excitability between the sham MEP(+) group and 1 Hz MEP(+) group (all *p* < 0.05). Furthermore, these patients with MEP(+) showed greater changes in the contralateral CMCT than the patients with MEP(−) after 1 Hz rTMS treatment (*t* = −3.304, *p* = 0.002). Nonetheless, no significant changes in the cortical excitability of bilateral hemispheres were found in these patients with no MEP at 8 weeks follow-up after 1 Hz rTMS treatment. Collectively, 1 Hz rTMS intervention could improve unaffected cortical excitability and inhibit affected cortical excitability of subacute stroke patients with MEP(+) compared to those patients with MEP(−) (Table 4).

**Figure 3:**
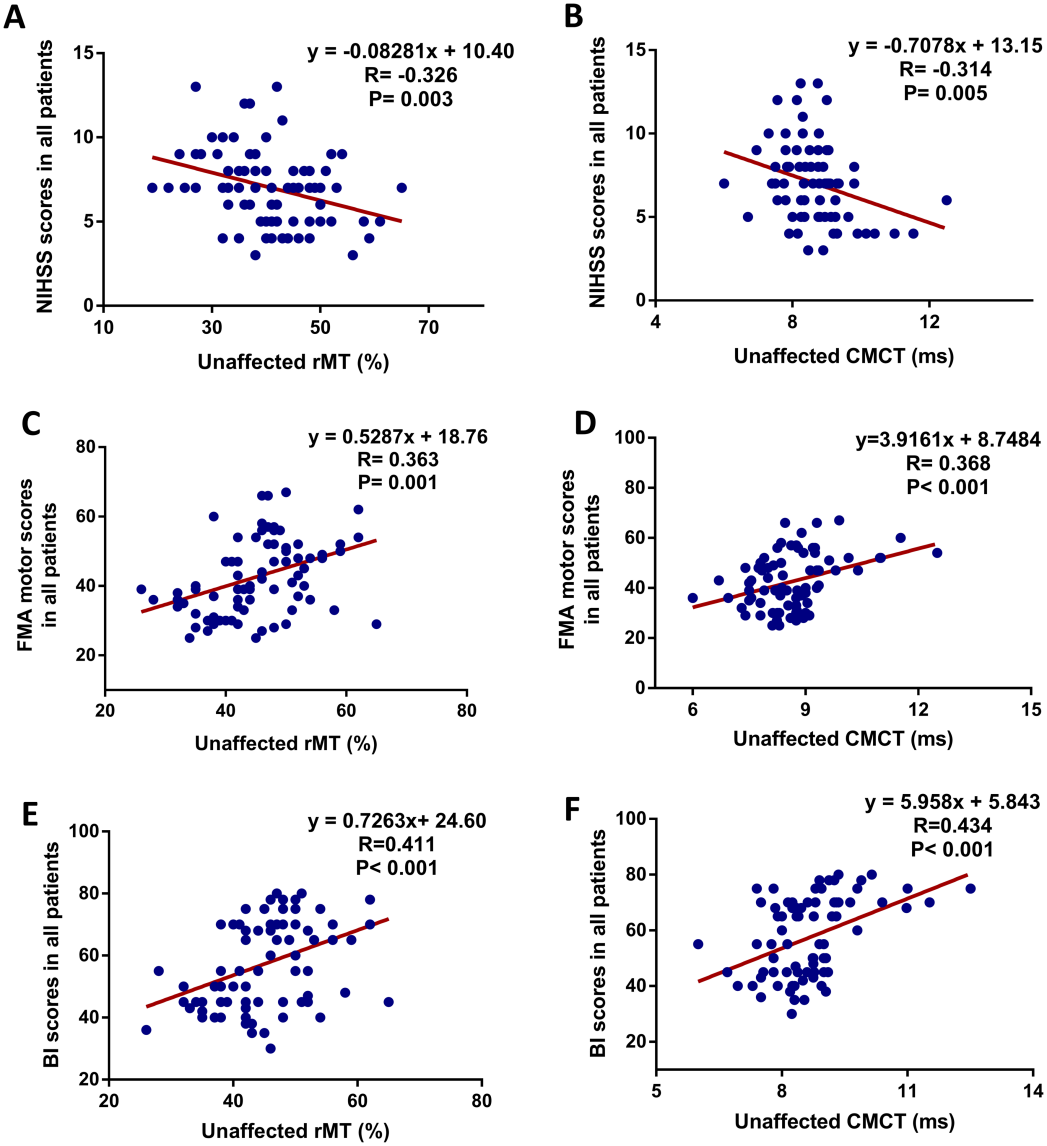
Scatter plot showing the relationship between motor function (increased FMA motor score) and unaffected cortical excitability in all patients at 8 weeks after intervention. **(A)** NIHSS scores and unaffected rMT, **(B)** NIHSS scores and unaffected CMCT, **(C)** FMA motor scores and unaffected rMT, **(D)** FMA motor scores and unaffected CMCT, **(E)** BI scores and unaffected rMT, **(F)** BI scores and unaffected CMCT.

**Figure 4:**
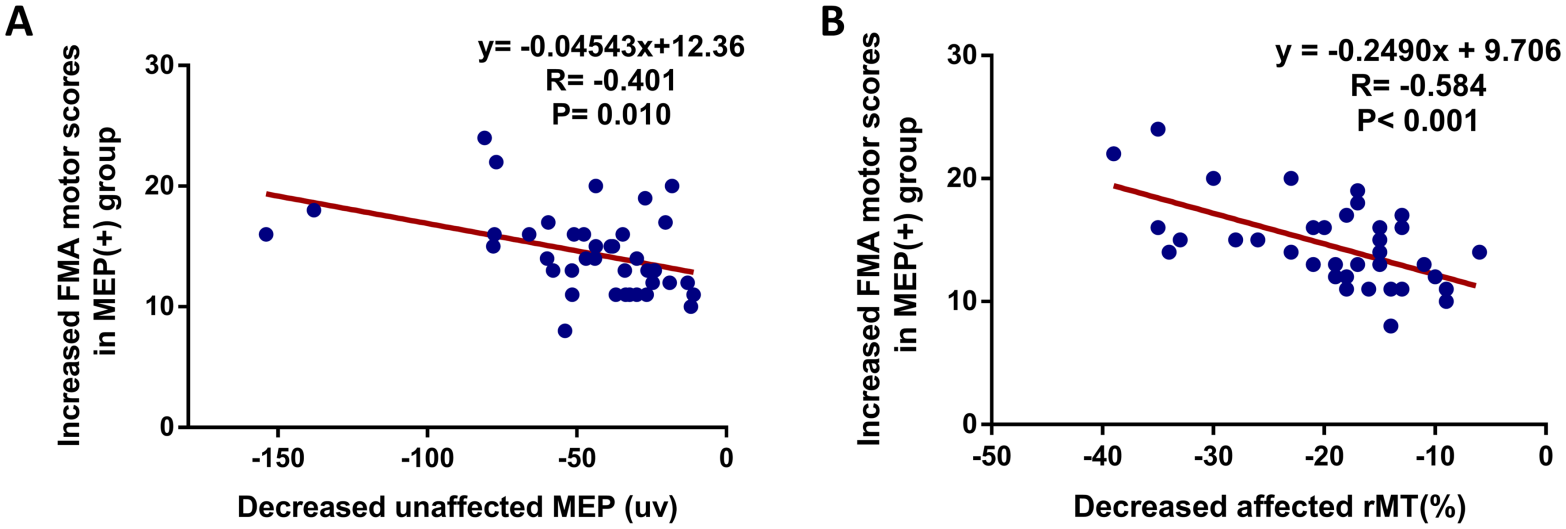
Scatter plot showing the relationship between motor function improvement (increased FMA motor score) and bilateral cortical excitability in MEP(+) group at 8 weeks after intervention. **(A)** Increased FMA motor score and decreased unaffected MEP amplitude, **(B)** increased FMA motor score and decreased affected rMT.

To explore the associations between motor function and motor cortex excitability at 8 weeks follow-up after interventions, statistical analysis showed a significant correlation between the NIHSS and unaffected rMT (*r* = −0.326, *p* = 0.003) (Figure 3A), NIHSS and unaffected CMCT (*r* = 0.314, *p* = 0.005) (Figure 3B), FMA and unaffected rMT (*r* = 0.363, *p* = 0.001) (Figure 3C), FMA and unaffected CMCT (*r* = 0.368, *p* < 0.001) (Figure 3D). Also, a positive association between the BI score and unaffected rMT (*r* = 0.411, *p* < 0.001) (Figure 3E), between the BI scores and unaffected CMCT (*r* = 0.434, *p* < 0.001) (Figure 3F) were observed. In addition, the associations between motor function improvement and motor cortex excitability changes at 8 weeks follow-up after interventions were further explored in this study. The correlation analysis revealed that FMA motor change score was associated with decreased unaffected MEP amplitude (*r* = −0.401, *p* = 0.010) and decreased affected rMT (*r* = −0.584, *p* < 0.001) from baseline, which was only observed in the MEP(+) group (Figure 4).

## Discussion

The current study aimed to demonstrate the effects of LF-rTMS on short-and long-term neurobehavioral and neurophysiology in subacute stroke patients with different MEP status. This study showed that LF-rTMS could improve motor function and regulate bilateral cortical excitability in subacute stroke patients with MEP, whereas LF-rTMS had no significant effects on motor function and bilateral cortical excitability in subacute stroke patients with no MEP. The results of the 8-week follow-up elucidated that LF-rTMS may be more effective in subacute stroke patients with MEP than in patients with no MEP. Moreover, the motor function improvement was closely related to the change of bilateral cortical excitability for these patients with MEP. Therefore, it is required for stroke patients to detect MEP status before rTMS treatment, which is beneficial to quickly evaluate the response of stroke patients to rTMS.

Based on recent advances in our understanding of brain plasticity and electrophysiological treatment strategies for stroke, rTMS has gradually been used to treat motor dysfunction after stroke (7). In various rTMS protocols, LF-rTMS has been applied to the contralateral M1 in attempts to normalize imbalanced interhemispheric inhibition (IHI) by suppressing the over-inhibition of the contralesional M1 toward the ipsilesional hemisphere (26). In addition, LF-rTMS is widely used in clinical treatment and in most studies due to the low risk of seizures (22). Although LF-rTMS is recommended for motor rehabilitation in subacute stroke patients (7), LF-rTMS treatment is not broadly used for all stroke patients. Many factors contribute to differences in the therapeutic effects of LF-rTMS, such as the duration of a stroke, severity of motor impairment, and hemispheric dominance (27). Considering subacute stage was the optimal time window for neuroplasticity and recovery after stroke, most studies selected these patients with subacute stroke. However, it is still contradictory that the effects of LF-rTMS on motor recovery in subacute stroke patients have been investigated in previous studies (8, 10, 16). Several studies reported that stroke patients receiving LF-rTMS had better motor recovery than sham controls (28, 29). In contrast, some studies reported that there was no significant improvement in motor function in stroke patients treated with low-frequency rTMS (27). In line with the above previous studies, we also found that not all stroke patients treated with LF-rTMS had better motor recovery compared to sham controls, as assessed by the NIHSS, FMA, SAFE, and BI at 4 and 8-week follow-up after intervention. In this study, only the stroke patients with MEP manifested great motor recovery after 1 Hz rTMS treatment, especially at the eight-week follow-up after treatment. In contrast, there were no significant changes in motor function after 1 Hz TMS treatment in stroke patients with no MEP. Therefore, these findings suggested that the response of stroke patients to LF-rTMS treatment may be related to the presence or absence of MEP after stroke.

The MEP evoked by TMS is a new and promising technique in the field of stroke rehabilitation. The use of MEP parameters is a non-invasive, and relatively safe technique and has the potential to provide information about the integrity of central motor pathways and motor cortical excitability (30). These parameters were also included as observational indicators in this study, such as rMT, MEP amplitude, CMCT, and IHA. The MEP has been used to assess the severity of brain injury and predict motor recovery in stroke patients (13, 15), and MEP status contribute to assessing the proportion of motor recovery in subacute stroke patients (13). However, previous studies have reported that there was a high false-negative incidence for MEP status in predicting motor recovery in the acute stage of stroke (31). MEP status in the subacute stage of stroke is more reliable for evaluating motor dysfunction or predicting of motor recovery (32). Therefore, we selected patients with subacute stroke to participate in this study. In our study, stroke patients with MEP manifested relatively lower severity of motor dysfunction than patients with no MEP, such as low NIHSS, high FMA, SAFE, and BI scores. This is consistent with previous studies showing that MEP status is related to motor recovery after stroke (33). Recently, the MEP has been widely used as a predictor of functional recovery and outcomes after stroke. However, it remains unknown whether MEP status is associated with response to rTMS in stroke patients. Our positive findings in stroke patients with the presence of MEP are consistent with some previous studies on subacute stroke with less severity of motor impairment (28, 34). However, the effects of LF-TMS on motor recovery of stroke patients with the absence of MEP were not significant, which is also consistent with previous studies on subacute stroke with severe motor dysfunction (35). Therefore, the effects of LF-TMS on motor recovery after stroke are closely related to the severity of stroke, and individual TMS treatment parameters should be considered based on the different severity of brain injury.

Recently, the theory of rTMS for the treatment of stroke is mainly based on the IHI model. The balance between the affected and unaffected hemispheres after stroke is disrupted, and it is usually manifested by reduced cortical excitability in the affected hemisphere and increased cortical excitability in the unaffected hemisphere (36), which were also detected by bilateral rMT, MEP amplitude, CMCT, and IHA of stroke patients with MEP in our study. Moreover, in these stroke patients with MEP, cortical excitability of unaffected hemisphere was effectively inhibited, while the cortical excitability of the affected hemisphere was significantly improved after the 4 weeks of LF-rTMS treatment. Nevertheless, no significant changes in cortical excitability were found after inhibitory LF-rTMS in stroke patients with no MEP, which is in consistent with previous studies (11, 37). Our results support the concept that inhibitory LF-rTMS of the contralesional motor cortex can reduce interhemispheric asymmetry, but only in these stroke patients with MEP. The changes in cortical excitability of those stroke patients with no MEP are still uncertain. The negative findings in stroke patients with no MEP can be explained by several reasons. First, the IHI model does not apply to all stroke patients (11, 38), such as stroke patients with no MEP. Second, the effects of LF-rTMS may vary depending on the severity of motor impairment. Recently, it has been proposed that a bimodal balance-recovery model can tailor individualized rTMS treatment based on brain injury severity and individual residual network. Our results also suggest that inhibition of the unaffected hemisphere by LF-rTMS may be more related to promoting motor recovery in patients with MEP than in patients with no MEP.

Research has demonstrated that the TMS-evoked MEP can be used to predict motor recovery and evaluate the extent of brain damage in stroke patients (13, 39). However, its role in determining the response of LF-rTMS on motor recovery remains to be further determined. To our knowledge, this is the first study to investigate the effects of LF-rTMS on motor recovery in stroke patients with different MEP status. Nevertheless, there were also some limitations to this study. The findings of this research can only be applied to patients who have similar clinical characteristics as those in our study, such as middle cerebral artery subacute stroke patients. The research was a single-center trial with a comparatively small sample size. To confirm the findings, a multicenter study with a larger sample size may be required. In addition, it is necessary to apply functional magnetic resonance imaging or near infrared functional imaging to provide more reliable and objective evidence.

## Conclusion

The main implications of this clinical trial are that the effects of LF-rTMS on motor recovery and regulating bilateral cortical excitability seems more effective in stroke patients with MEP than those with no MEP. The findings revealed that the response of stroke patients to LF-rTMS was related to MEP status after stroke. Moreover, the presence of MEP may suggest that LF-rTMS is effective in improving motor function and regulating bilateral cortical excitability after stroke. Therefore, electrophysiological detection of MEP status in stroke patients before rTMS treatment is beneficial to evaluate rTMS responsiveness and therapeutic effects. In the future, it is recommended to conduct electrophysiological evaluations before rTMS treatment for stroke patients to achieve effective therapeutic effects, such as MEP status.

## Data Availability

The raw data supporting the conclusions of this article will be made available by the authors, without undue reservation.

## References

[1] Collaborators GBDA. The burden and trend of diseases and their risk factors in Australia, 1990-2019: a systematic analysis for the global burden of disease study 2019. Lancet Public Health. (2023) 8:e585–99. doi: 10.1016/S2468-2667(23)00123-8 37516475 PMC10400798

[2] Hilkens NA Casolla B Leung TW de Leeuw FE. Stroke. Lancet. (2024) 403:2820–36. doi: 10.1016/S0140-6736(24)00642-1 38759664

[3] Tu WJ Zhao Z Yin P Cao L Zeng J Chen H. Estimated burden of stroke in China in 2020. JAMA Netw Open. (2023) 6:e231455. doi: 10.1001/jamanetworkopen.2023.1455, PMID: 36862407 PMC9982699

[4] Mi Y Huai L Yin Y Yuan J Liu Y Huang J. Burden of stroke in China and the different SDI regions over the world. J Glob Health. (2023) 13:04169. doi: 10.7189/jogh.13.04169, PMID: 38131457 PMC10740341

[5] Bernhardt J Hayward KS Kwakkel G Ward NS Wolf SL Borschmann K. Agreed definitions and a shared vision for new standards in stroke recovery research: the stroke recovery and rehabilitation roundtable taskforce. Int J Stroke. (2017) 12:444–50. doi: 10.1177/1747493017711816, PMID: 28697708

[6] Raffin E Hummel FC. Restoring motor functions after stroke: multiple approaches and opportunities. Neuroscientist. (2018) 24:400–16. doi: 10.1177/1073858417737486, PMID: 29283026

[7] Lefaucheur JP Aleman A Baeken C Benninger DH Brunelin J Di Lazzaro V. Evidence-based guidelines on the therapeutic use of repetitive transcranial magnetic stimulation (rTMS): An update (2014-2018). Clin Neurophysiol. (2020) 131:474–528. doi: 10.1016/j.clinph.2019.11.002, PMID: 31901449

[8] Kim WS Kwon BS Seo HG Park J Paik NJ. Low-frequency repetitive transcranial magnetic stimulation over Contralesional motor cortex for motor recovery in subacute ischemic stroke: a randomized sham-controlled trial. Neurorehabil Neural Repair. (2020) 34:856–67. doi: 10.1177/1545968320948610, PMID: 32807013

[9] Wang Q Zhang D Zhao YY Hai H Ma YW. Effects of high-frequency repetitive transcranial magnetic stimulation over the contralesional motor cortex on motor recovery in severe hemiplegic stroke: a randomized clinical trial. Brain Stimul. (2020) 13:979–86. doi: 10.1016/j.brs.2020.03.020, PMID: 32380449

[10] Huang YZ Lin LF Chang KH Hu CJ Liou TH Lin YN. Priming with 1-Hz repetitive transcranial magnetic stimulation over Contralesional leg motor cortex does not increase the rate of regaining ambulation within 3 months of stroke: a randomized controlled trial. Am J Phys Med Rehabil. (2018) 97:339–45. doi: 10.1097/PHM.0000000000000850, PMID: 29023249

[11] Di Pino G Pellegrino G Assenza G Capone F Ferreri F Formica D. Modulation of brain plasticity in stroke: a novel model for neurorehabilitation. Nat Rev Neurol. (2014) 10:597–608. doi: 10.1038/nrneurol.2014.162, PMID: 25201238

[12] McDonnell MN Stinear CM. TMS measures of motor cortex function after stroke: a meta-analysis. Brain Stimul. (2017) 10:721–34. doi: 10.1016/j.brs.2017.03.008, PMID: 28385535

[13] Stinear CM. Prediction of motor recovery after stroke: advances in biomarkers. Lancet Neurol. (2017) 16:826–36. doi: 10.1016/S1474-4422(17)30283-1, PMID: 28920888

[14] Talelli P Greenwood RJ Rothwell JC. Arm function after stroke: neurophysiological correlates and recovery mechanisms assessed by transcranial magnetic stimulation. Clin Neurophysiol. (2006) 117:1641–59. doi: 10.1016/j.clinph.2006.01.016 16595189

[15] Karatzetzou S Tsiptsios D Terzoudi A Aggeloussis N Vadikolias K. Transcranial magnetic stimulation implementation on stroke prognosis. Neurol Sci. (2022) 43:873–88. doi: 10.1007/s10072-021-05791-1, PMID: 34846585

[16] Luk KY Ouyang HX Pang MYC. Low-frequency rTMS over Contralesional M1 increases Ipsilesional cortical excitability and motor function with decreased interhemispheric asymmetry in subacute stroke: a randomized controlled study. Neural Plast. (2022) 2022:1–13. doi: 10.1155/2022/3815357 PMC8756161 35035473

[17] van Lieshout ECC van der Worp HB Visser-Meily JMA Dijkhuizen RM. Timing of repetitive transcranial magnetic stimulation onset for upper limb function after stroke: a systematic review and Meta-analysis. Front Neurol. (2019) 10:1269. doi: 10.3389/fneur.2019.01269, PMID: 31849827 PMC6901630

[18] Choi EY Nieves GA Jones DE. Acute stroke diagnosis. Am Fam Physician. (2022) 105:616–24. PMID: 35704804

[19] Vucic S Stanley Chen KH Kiernan MC Hallett M Benninger DH Di Lazzaro V. Clinical diagnostic utility of transcranial magnetic stimulation in neurological disorders. Updated report of an IFCN committee. Clin Neurophysiol. (2023) 150:131–75. doi: 10.1016/j.clinph.2023.03.010, PMID: 37068329 PMC10192339

[20] van Kuijk AA Pasman JW Hendricks HT Zwarts MJ Geurts AC. Predicting hand motor recovery in severe stroke: the role of motor evoked potentials in relation to early clinical assessment. Neurorehabil Neural Repair. (2009) 23:45–51. doi: 10.1177/1545968308317578, PMID: 18794218

[21] Gladstone DJ Danells CJ Black SE. The fugl-meyer assessment of motor recovery after stroke: a critical review of its measurement properties. Neurorehabil Neural Repair. (2002) 16:232–40. doi: 10.1177/154596802401105171, PMID: 12234086

[22] Payabvash S Taleb S Benson JC McKinney AM. Acute ischemic stroke infarct topology: association with lesion volume and severity of symptoms at admission and discharge. AJNR Am J Neuroradiol. (2017) 38:58–63. doi: 10.3174/ajnr.A4970, PMID: 27758775 PMC7963653

[23] Kobayashi M Pascual-Leone A. Transcranial magnetic stimulation in neurology. Lancet Neurol. (2003) 2:145–56. doi: 10.1016/S1474-4422(03)00321-1 12849236

[24] Chen R Cros D Curra A Di Lazzaro V Lefaucheur JP Magistris MR. The clinical diagnostic utility of transcranial magnetic stimulation: report of an IFCN committee. Clin Neurophysiol. (2008) 119:504–32. doi: 10.1016/j.clinph.2007.10.014, PMID: 18063409

[25] Mishory A Molnar C Koola J Li X Kozel FA Myrick H. The maximum-likelihood strategy for determining transcranial magnetic stimulation motor threshold, using parameter estimation by sequential testing is faster than conventional methods with similar precision. J ECT. (2004) 20:160–5. doi: 10.1097/00124509-200409000-00007 15343000

[26] Zhang L Xing G Fan Y Guo Z Chen H Mu Q. Short-and long-term effects of repetitive transcranial magnetic stimulation on upper limb motor function after stroke: a systematic review and Meta-analysis. Clin Rehabil. (2017) 31:1137–53. doi: 10.1177/0269215517692386, PMID: 28786336

[27] Ludemann-Podubecka J Bosl K Theilig S Wiederer R Nowak DA. The effectiveness of 1 Hz rTMS over the primary motor area of the unaffected hemisphere to improve hand function after stroke depends on hemispheric dominance. Brain Stimul. (2015) 8:823–30. doi: 10.1016/j.brs.2015.02.004 25828427

[28] Du J Tian L Liu W Hu J Xu G Ma M. Effects of repetitive transcranial magnetic stimulation on motor recovery and motor cortex excitability in patients with stroke: a randomized controlled trial. Eur J Neurol. (2016) 23:1666–72. doi: 10.1111/ene.13105 27425785

[29] Li J Meng XM Li RY Zhang R Zhang Z Du YF. Effects of different frequencies of repetitive transcranial magnetic stimulation on the recovery of upper limb motor dysfunction in patients with subacute cerebral infarction. Neural Regen Res. (2016) 11:1584–90. doi: 10.4103/1673-5374.193236 27904488 PMC5116836

[30] Bai Z Zhang J Fong KNK. Effects of transcranial magnetic stimulation in modulating cortical excitability in patients with stroke: a systematic review and meta-analysis. J Neuroeng Rehabil. (2022) 19:24. doi: 10.1186/s12984-022-00999-4, PMID: 35193624 PMC8862292

[31] Lundquist CB Nielsen JF Arguissain FG Brunner IC. Accuracy of the upper limb prediction algorithm PREP2 applied 2 weeks Poststroke: a prospective longitudinal study. Neurorehabil Neural Repair. (2021) 35:68–78. doi: 10.1177/1545968320971763, PMID: 33218284

[32] Kwon YM Jang SH Lee JW. Predictability of motor outcome according to the time of motor evoked potentials from the onset of stroke in patients with Putaminal hemorrhage. Ann Rehabil Med. (2015) 39:553–9. doi: 10.5535/arm.2015.39.4.553, PMID: 26361591 PMC4564702

[33] Lai CJ Wang CP Tsai PY Chan RC Lin SH Lin FG. Corticospinal integrity and motor impairment predict outcomes after excitatory repetitive transcranial magnetic stimulation: a preliminary study. Arch Phys Med Rehabil. (2015) 96:69–75. doi: 10.1016/j.apmr.2014.08.014, PMID: 25218256

[34] Du J Yang F Hu J Hu J Xu Q Cong N. Effects of high- and low-frequency repetitive transcranial magnetic stimulation on motor recovery in early stroke patients: evidence from a randomized controlled trial with clinical, neurophysiological and functional imaging assessments. Neuroimage Clin. (2019) 21:101620. doi: 10.1016/j.nicl.2018.101620, PMID: 30527907 PMC6411653

[35] Tosun A Ture S Askin A Yardimci EU Demirdal SU Kurt Incesu T. Effects of low-frequency repetitive transcranial magnetic stimulation and neuromuscular electrical stimulation on upper extremity motor recovery in the early period after stroke: a preliminary study. Top Stroke Rehabil. (2017) 24:361–7. doi: 10.1080/10749357.2017.1305644, PMID: 28327054

[36] Jannati A Oberman LM Rotenberg A Pascual-Leone A. Assessing the mechanisms of brain plasticity by transcranial magnetic stimulation. Neuropsychopharmacology. (2023) 48:191–208. doi: 10.1038/s41386-022-01453-8, PMID: 36198876 PMC9700722

[37] Dodd KC Nair VA Prabhakaran V. Role of the Contralesional vs. Ipsilesional hemisphere in stroke recovery. Front Hum Neurosci. (2017) 11:469. doi: 10.3389/fnhum.2017.00469, PMID: 28983244 PMC5613154

[38] Di Pino G Di Lazzaro V. The balance recovery bimodal model in stroke patients between evidence and speculation: do recent studies support it? Clin Neurophysiol. (2020) 131:2488–90. doi: 10.1016/j.clinph.2020.07.004, PMID: 32747189

[39] Pizzi A Carrai R Falsini C Martini M Verdesca S Grippo A. Prognostic value of motor evoked potentials in motor function recovery of upper limb after stroke. J Rehabil Med. (2009) 41:654–60. doi: 10.2340/16501977-0389, PMID: 19565160

